# Evidence for impaired clearance leading to amyloid beta (Aβ) accumulation in the Alzheimer’s eye

**DOI:** 10.1002/alz.087166

**Published:** 2025-01-03

**Authors:** Printha Wijesinghe, Amir Hosseini, Matthew Campbell, Jeanne Xi, Justin R Haynes, Ian R MacKenzie, Veronica Hirsch‐Reinshagen, Ging‐Yuek Robin Hsiung, Benjamin Spiller, Brian E Wadzinski, Wellington Pham, Joanne A Matsubara

**Affiliations:** ^1^ The University of British Columbia, Vancouver, BC Canada; ^2^ Vanderbilt University Medical Center, Nashville, TN USA; ^3^ Djavad Mowafaghian Centre for Brain Health, Vancouver, BC Canada

## Abstract

**Background:**

An imbalance between the production and clearance of amyloid beta (Aß) has emerged as a major cause of sporadic Alzheimer’s disease (AD). Retinal wholemount studies can identify cell‐specific involvement in Aß clearance mechanisms which cannot be accomplished in the brain *ex vivo*.

**Methods:**

Eye cross‐sections of double transgenic (Tg, APP‐PS1) and non‐carrier sibling female mice (n = 16, 4 per group) at 3‐ and 9‐ month ages were probed with antibodies 6E10 (Aβ1‐16 amino‐acid residues, soluble and insoluble species), ionized calcium‐binding adapter molecule 1 (IBA1, microglia/macrophage), glial fibrillary acidic protein (GFAP, astrocytes), glutamine synthetase (GS, Müller cells) and aquaporin‐4 (AQP4, membrane water channel) using immunofluorescence. Wholemount neuroretinas of human AD (n = 10, mean age = 76.8, 6 females) and control (n = 10, 72.5, 5 females) donor eyes were probed with antibodies 12F4 (Aß1‐42 amino‐acid residues, misfolded oligomers), IBA1, GFAP, GS, AQP4 and Ulex europaeus agglutinin (UEA‐l, vascular endothelium). Finally, a nanobody (soluble Aß oligomer against human Aβ1‐42 peptide) conjugated with near‐infrared dye (NIR‐E3) was probed with 12F4 using a nanobody staining protocol in wholemount retinas.

**Results:**

In eye cross‐sections, a significant increase in 6E10, IBA1, GFAP and AQP4 was found in the older *vs*. younger Tg mice, suggesting Aβ accumulation resulted in gliosis and AQP4 mislocalization. In contrast, a significant reduction for 6E10 in the older *vs*. younger non‐carrier siblings, suggests the existence of an effective Aβ clearance mechanism (e.g., macrophages and glymphatic drainage) as IBA1 and AQP4 levels were increased. In wholemount neuroretinas, macroglia degenerations (GFAP, GS and AQP4) which form the inner limiting membrane were prominent in human AD eyes, suggesting defective ocular glymphatic systems. The microglia phenotype was different from its resting stage in AD eyes, however, due to complexity of IBA1 positive resident microglia, and macrophages including monocytes, perivascular macrophages, pericytes and hyalocytes, it is difficult to define microglia phenotype, particularly activated and ameboid types between AD and control eyes. Interestingly, NIR‐E3 positive perivascular macrophages (but negative to 12F4), indicating soluble Aβ clearance were predominantly found within the blood vessels of control donor eyes.

**Conclusion:**

This study confirms the existence of different impaired clearance mechanisms in human AD eyes.

